# Editorial: Animal models of hypertension

**DOI:** 10.3389/fphys.2025.1542502

**Published:** 2025-01-13

**Authors:** Marta Aleksandrowicz, Marta Kuczeriszka, Leszek Dobrowolski, Modar Kassan

**Affiliations:** ^1^ Laboratory of Preclinical Research and Environmental Agents, Mossakowski Medical Research Institute, Polish Academy of Sciences, Warsaw, Poland; ^2^ Department of Renal and Body Fluid Physiology, Mossakowski Medical Research Institute, Polish Academy of Sciences, Warsaw, Poland; ^3^ College of Dental Medicine, Lincoln Memorial University, Harrogate, TN, United States

**Keywords:** hypertension, animal models, pathogenesis, physical exercise, trimethylamine oxide, nitric oxide, metabolic syndrome

One of the first choices that researchers must make when selecting the best model of hypertension is whether to use small or large animal models. However, a literature review indicates that the vast majority of experimental studies are performed on rodents. Several rodent models have been proposed to mimic the hypertension pathogenesis observed in humans. They can be divided into the following models: 1) genetically-induced: Spontaneously Hypertensive Rat (SHR); stroke-prone SHR; Dahl Salt-Sensitive Rats, 2) pharmacologically-induced: Angiotensin II (ANG II)-induced hypertension; deoxycorticosterone acetate (DOCA)-salt-induced hypertension; high salt diet (HSD)-induced hypertension; N-nitro-L-arginine methyl ester (L-NAME)-induced hypertension; fructose-induced hypertension, and 3) surgically induced: the two-kidney one-clip model (i.e., constriction of only one renal artery), the two-kidney two-clip model (i.e., aortic constriction or constriction of both renal arteries), or the one-kidney one-clip model (i.e., constriction of one renal artery and ablation of the contralateral kidney). The separate disease unit is pulmonary hypertension, which is induced in animals mainly pharmacologically (monocrotaline, bleomycin), surgically, or by hypoxia. Each model offers distinct advantages for studying various aspects of hypertension; therefore, selecting the appropriate animal model is essential depending on the studied mechanism.

The articles on this Research Topic were collected from studies performed in various animal models of hypertension. They advance our current knowledge in the pathogenesis and treatment of hypertension, focusing mainly on the beneficial effects of physical exercise, differences in gene and protein expression between animal models, and mechanisms involved in the neural control of cardiovascular functions.

In the paper by Drummond et al., pulmonary arterial hypertension was induced in Wistar rats by a single intraperitoneal injection with monocrotaline in a dose of 60 mg/kg. The study results showed that voluntary running prevented the increase in pulmonary artery resistance, increased respiratory function, and generally benefited the rat’s survival and exercise tolerance. These favorable effects were associated with increased muscle fiber and increased expression of genes involved in mitochondrial biogenesis in the skeletal muscle of rats with monocrotaline-induced pulmonary arterial hypertension.

The positive effect of physical exercise is also related to its involvement in cardiac and skeletal muscle angiogenesis. Angiogenesis, the formation of blood vessels from the existing vasculature, is known to improve blood flow and decrease vascular resistance. The studies of Macedo et al. were performed in spontaneously hypertensive rats (SHR) and concerned which drug, captopril or perindopril, would be better in treating hypertension in the context of the positive effect of exercise-induced angiogenesis. Both drugs are inhibitors of angiotensin-converting enzyme (ACE), which are very effective in reducing blood pressure but are known to attenuate exercise-induced angiogenesis. In this article, rat treatment with captopril attenuated training-induced angiogenesis, but what is surprising is that after perindopril treatment in SHR, angiogenesis was less diminished. Results like these indicate that perindopril is a better medication for hypertensive individuals who engage in aerobic exercise.

The subsequent studies published on this Research Topic by Gawryś-Kopczyńska et al. were performed in two animal models of hypertension: on SHRs and ANG II-induced hypertensive rats. ANG II was administered by subcutaneously implanted osmotic minipump. This paper concerns whether different models of hypertension and, consequently, different blood pressure phenotypes in rats could influence variations in the expression of flavin monooxygenase (FMO), an enzyme that oxidizes trimethylamine (TMA) to trimethylamine oxide (TMAO). In turn, TMAO is a significant risk factor for cardiovascular diseases. The study results demonstrated that SHRs, in contrast to ANG-induced hypertensive rats, showed elevated hepatic gene expression and protein levels of FMOs and more rapid oxidation of TMA to TMAO. This study demonstrates how crucial it is to select the appropriate hypertension model to capture the investigated pathophysiological mechanism and to interpret the findings more comprehensively. Thus, conducting experimental studies in more than one model may be beneficial for investigating some research hypotheses.

Finally, the paper by Zhu et al. concerned the neural control of cardiovascular functions in the model of fructose-induced hypertension. On the one hand, in this model, fructose stimulates sodium and chloride absorption, leading to salt overload, which raises blood pressure. On the other hand, long-term feeding of animals with fructose leads to hyperglycemia, hypertriglyceridemia, and insulin resistance. Thus, the metabolic changes in fructose-fed rats resemble human metabolic syndrome. The study by Zhu et al. aimed to assess if nitric oxide (NO) is involved in the synaptic plasticity underlying the elevated sympathetic outflow in fructose-induced hypertension. The study showed that fructose feeding reduced NO production, NO-induced glutamate release in the nucleus tractus solitarius, and depressor response.

Studies published in this Research Topic were performed in various animal models of hypertension. They provide insights into the complex mechanisms underlying hypertension and help to identify new actionable therapeutic targets. However, translating results from animals to humans requires careful consideration, as there are species-specific differences in blood pressure regulation and response to therapies. Moreover, no single model fully recapitulates the complexity of human hypertension. Nevertheless, animal models, mainly rodents, remain fundamental in developing strategies to combat hypertension and its complications.

